# TGF-β-Driven Atrial Fibrosis in Atrial Fibrillation: From Mechanistic Insights to Targeted Therapies

**DOI:** 10.14336/AD.2025.0564

**Published:** 2026-07-27

**Authors:** Chen Chen, Pengfei Chen, Wenxi Yu, Lingli Zhao, Yu Yang, Hua Qu, Changgeng Fu, Dazhuo Shi, Ming Guo

**Affiliations:** ^1^Xiyuan Hospital, China Academy of Chinese Medical Sciences, Beijing, China.; ^2^Cardiovascular Diseases Center, Xiyuan Hospital, China Academy of Chinese Medical Sciences, Beijing, China.; ^3^Department of Graduate School, Beijing University of Chinese Medicine, Beijing, China.

**Keywords:** TGF-β, Atrial Fibrillation, Atrial Fibrosis, Targeted Therapies

## Abstract

The prevalence of atrial fibrillation (AF), one of the most common cardiac arrhythmias, has significantly increased, especially within the aging population. Atrial fibrosis, a hallmark of the structural changes seen in AF, is characterized by abnormal activation and proliferation of atrial fibroblasts accompanied by excessive deposition of extracellular matrix. The initiation and progression of fibrosis are regulated by multiple profibrotic cytokines, with transforming growth factor-beta (TGF-β) serving as the primary mediator. Elevated expression and activation of TGF-β are frequently observed in myocardial diseases, where it drives myofibroblast differentiation, migration, and collagen production via the Smad-dependent and Smad-independent pathways. These fibrotic alterations disrupt normal atrial electrical conduction, thereby facilitating the onset and progression of AF. This review summarizes the role of TGF-β-mediated fibrotic remodeling in AF pathogenesis and treatment. We explored the molecular mechanisms underlying TGF-β signaling in atrial fibrosis, evaluated its potential as a predictive biomarker for AF and recurrence after ablation, and discussed therapeutic strategies targeting this pathway. Increasing evidence from studies on natural and synthetic TGF-β inhibitors highlights the potential of modulating this signaling cascade to attenuate fibrosis and enhance clinical outcomes. Our review underscores the pivotal role of TGF-β signaling in AF development and supports its potential as a promising target for therapeutic intervention capable of improving the efficacy of antiarrhythmic treatments.

## 1. Introduction

In 2023, approximately 80 million individuals in the Asia-Pacific region were affected by atrial fibrillation (AF) [1]. With the aging of global population, increasing prevalence of comorbid conditions, and advancements in diagnostic technologies alongside heightened clinical awareness, the incidence of AF is expected to continue increasing [2, 3]. As a major risk factor for myocardial infarction (MI), heart failure, stroke, and cognitive decline, AF imposes a substantial burden on healthcare systems by increasing medical resource utilization and associated costs [4]. According to research conducted using the Optum Clinformatics database, the annual health care expenses for individuals with AF reach $63,031, exceeding the costs for those without AF by $27,896 [5]. Current guideline-recommended treatments for AF include antiarrhythmic drugs (AADs), anticoagulation therapy, and catheter ablation (CA). However, the clinical use of traditional AADs and anticoagulants is often limited by their adverse effects, such as proarrhythmic risks, dose-dependent gastro-intestinal complications, and potentially fatal intracranial hemorrhages. Despite key advances in CA-based treatments, this treatment shows limited or no benefit in individuals with specific AF types, those with structural remodeling (such as left atrial dilatation or atrial/ventricular fibrosis), or those with multiple comorbid conditions [6, 7]. Among the various contributing factors, left atrial fibrosis is particularly notable as it is a marker of structural remodeling as well as a predictor of AF recurrence after CA. In addition, left atrial fibrosis is a key driver in the pathophysiological progression of AF [8, 9]. Areas of fibrosis are characterized by disrupted and non-uniform atrial electrical conduction, a condition that facilitates the initiation and persistence of AF [10]. Therefore, interventions aimed at modulating upstream pathways of cardiac fibrosis may enhance the therapeutic effectiveness of AADs and help prevent the development of AF.

Transforming growth factor-β (TGF-β) is a pleiotropic cytokine involved in the regulation of diverse cellular functions, such as maintaining immune balance, guiding embryonic development, controlling programmed cell death, and promoting or inhibiting cell growth [11]. TGF-β signaling mediates a wide range of biological effects via the Smad-dependent and Smad-independent pathways, and TGF-β’s activity is further regulated by coreceptors and complex molecular networks [12]. Notably, TGF-β is widely recognized as a central regulator of cardiac remodeling and tissue repair. It influences key processes such as fibroblast activation, extracellular matrix production, cell proliferation and migration, epithelial-to-mesenchymal transition, and immune response regulation [13]. Clinical research has also revealed that higher serum levels of TGF-β are associated with more pronounced cardiac fibrosis and a higher risk of AF recurrence after surgical maze intervention [14]. Targeting TGF-β directly or through its signaling pathway has proven efficient in reducing cardiac fibrosis. For instance, galunisertib, a TGF-β-specific inhibitor currently under clinical trial for various cancer types, has shown promise in mitigating myocardial fibrosis, thereby contributing to MI treatment and facilitating cardiac tissue repair [15]. Current evidence suggests that TGF-β-driven atrial fibrosis plays a significant role in the development of arrhythmias, highlighting TGF-β as a promising therapeutic target in AF.

Given TGF-β’s central role in fibrosis, strategies designed to inhibit its activation or disrupt its downstream signaling pathways are being extensively studied, with the goal of mitigating or potentially reversing fibrosis and its related pathological consequences [16]. Several therapeutic strategies targeting TGF-β have been proposed for AF, primarily due to their antifibrotic effects. In recent years, multiple small-molecule agents have been developed to modulate TGF-β signaling pathways. This review summarizes the molecular mechanisms by which TGF-β promotes atrial fibrosis in AF and assesses the therapeutic potential of targeting this pathway in clinical settings. Furthermore, it explores emerging perspectives that may inform the development of innovative strategies for the prevention and treatment of fibrotic cardiac diseases.

## 2. Atrial Fibrosis in AF

**Figure 1.**
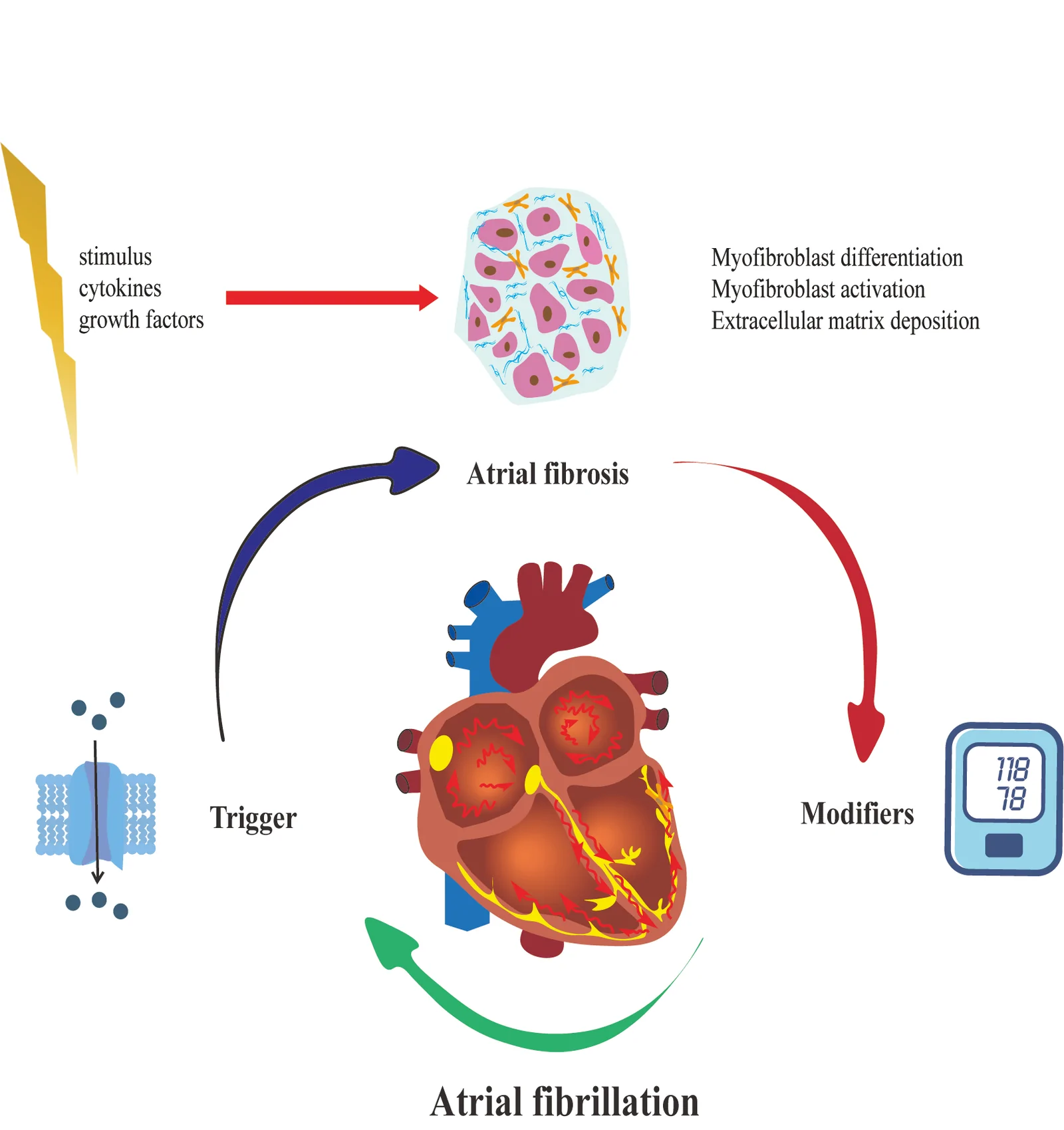
Mechanisms of atrial fibrosis in atrial fibrillation.

The pathogenesis of AF encompasses three main stages: initiation, maintenance, and progression toward chronic or persistent forms [17]. As illustrated in Figure 1, atrial fibrosis, a prominent feature of structural remodeling, plays an essential role in the development and advancement of AF, contributing to the initial onset and further progression of atrial fibrosis.

### 2.1. Mechanisms of Atrial Fibrosis

Fibrosis involves the abnormal accumulation of extracellular matrix (ECM) proteins, which serve a protective role in the early phases and are essential for tissue repair and regeneration [18]. In normal tissues, fibroblasts are responsible for maintaining the integrity of the ECM and are crucial for regulating collagen synthesis and degradation. However, under pathological conditions, excessive ECM accumulation increases tissue rigidity and disrupts intercellular connectivity, thereby impairing contractile function and signal transmission [19].

Cardiac fibrosis is a common pathological feature observed across various cardiovascular conditions, including those associated with diabetes, ischemic injury, advanced age, and genetic cardiomyopathies. These conditions are accompanied by inflammation and oxidative stress that influence fibroblast activation and pathological remodeling progression. Transient activation of myofibroblasts aids tissue repair, but persistent fibrotic responses can lead to cardiac structural distortion and impaired cardiac function, leading to increased myocardial stiffness and impaired relaxation and contraction capabilities. Although increased tissue stiffness is a direct outcome of fibrotic remodeling, it also acts as a strong stimulus that activates fibroblasts and drives their differentiation into myofibroblasts [20]. The inherent mechanosensitivity of fibroblasts is governed by several key signaling pathways, including TGF-β, yes-associated protein (YAP), and transcriptional co-activator with PDZ-binding motif (TAZ). These pathways establish a self-amplifying loop in which the rigid ECM persistently promotes fibroblast activation and fibrogenesis, even after the original pro-fibrotic and inflammatory triggers have subsided [21, 22]. Consequently, fibrosis can be conceptualized as a sustained cycle of aberrant tissue repair and remodeling, which over time evolves into a persistent inflammatory and degenerative condition, eventually culminating in irreversible fibrotic changes.

### 2.2. Arrhythmogenic Effects of Atrial Fibrosis in AF

Histopathologically, cardiac fibrosis is generally categorized into three distinct forms: replacement fibrosis, interstitial fibrosis, and perivascular fibrosis [23]. The resultant myocardial disorganization and altered cellular composition promotes arrhythmogenesis through multiple mechanisms [24]. A common feature of all distinct forms of cardiac fibrosis is mechanical stiffness caused by excessive deposition of ECM [25]. First, accumulation of ECM components and collagen can weaken electrical coupling between cardiomyocytes, thereby promoting localized ectopic activity. This may lead to abnormal electrical impulses originating in the atria, which can override the sinoatrial node and interfere with normal rhythm regulation, ultimately triggering arrhythmias. Atrial fibrosis further drives structural remodeling within the heart, leading to increased myocardial stiffness and impaired ventricular relaxation and contractility. The resulting mechanical stress can stimulate additional integrin activation, facilitating the release of TGF-β from its latent complexes and initiating a mechanotransduction-driven positive feedback loop that promotes fibroblast differentiation [26]. Consequently, fibroblasts transform into myofibroblasts, which actively proliferate and secrete ECM components, such as collagen. These myofibroblasts also express contractile and signaling proteins, including α-smooth muscle actin (α-SMA), mechanosensitive ion channels, and connexins, thereby playing a critical role in the development and recurrence of AF.

### 2.3. Clinical Association Between Atrial Fibrosis and AF

Left atrial fibrosis, in particular, has emerged as a significant severity predictor for the onset of AF and the overall burden of arrhythmias. Consistent histopathological findings have confirmed the frequent occurrence of atrial fibrosis in patients with AF [27-29], and its severity has been shown to correlate positively with the duration of the condition [30]. While CA is widely regarded as an effective method for achieving rhythm control, pulmonary vein isolation as a standalone procedure has been shown to be relatively less effective in patients with persistent AF than in those with paroxysmal AF [31]. A retrospective study involving 426 patients with a 1-year follow-up revealed a strong association between the extent of left atrial fibrosis and the incidence of arrhythmia recurrence. The recurrence rates for stages I, II, III, and IV fibrosis were 21.0%, 29.3%, 33.8%, and 71.4%, respectively [32]. Another retrospective study involving 308 patients with a 5-year follow-up reported a strong association between the extent of left atrial fibrosis and the long-term outcomes of AF ablation. Patients with more pronounced atrial fibrosis were more likely to experience recurrence of AF and underwent repeat ablation procedures at a higher rate [33]. Treatments targeting atrial fibrosis aim to reduce or reverse this process, potentially improving AF management.

### 2.4. Current Treatment Limitations

Therapeutically, while various antifibrotic agents targeting established profibrotic pathways have shown encouraging results, robust evidence supporting their sustained effectiveness in reducing atrial fibrosis and improving patient outcomes is still lacking. For instance, experimental studies in different disease models have reported that sodium-glucose cotransporter 2 (SGLT2) inhibitors and glucagon-like peptide-1 receptor agonists can reduce cardiac fibrosis, but their efficacy in treating AF has been modest in clinical trials [34, 35]. Furthermore, epigenetic mechanisms, including histone modification, DNA methylation, and chromatin remodeling, have been recognized as pivotal in the onset and progression of fibrosis. Besides traditional pharmacological agents, numerous microRNAs (miRNAs) are capable of modulating abnormal TGF-β signaling, acting as post-transcriptional regulators of genes implicated in cardiac fibrosis [36]. Collectively, these epigenetic modifications hold substantial promise for the treatment of fibrotic diseases and their application in biotechnological innovation.

In summary, atrial fibrosis compromises the structural organization of cardiac tissue and disrupts the electrophysiological function of cardiomyocytes by impairing intercellular communication and paracrine signaling, thus constituting a critical pathogenic factor in the onset of AF (Fig. 1). Accumulating evidence supports the notion that targeted therapies can partially reverse atrial remodeling; however, effective and specific agents that directly target myocardial fibrosis are still scarce. Therefore, strategies aimed at mitigating fibrosis may improve clinical outcomes for patients with AF.

## 3. TGF-β is a Predictive Biomarker for AF

Since invasive mapping techniques are rarely utilized in AF cases, circulating biomolecules may serve as quantitative indicators of cardiovascular disease progression, even in the early, subclinical stages. The selection of appropriate therapeutic strategies and prediction of the long-term success of CA largely rely on evaluating arrhythmogenic risk factors, with fibrosis being of particular importance.

A recent study reported that TGF-β1 expression levels were significantly higher in patients with chronic AF than in those with persistent AF, based on tissue samples obtained from the atrial appendages [37]. A clinical investigation involving 16 patients with chronic AF who underwent CA demonstrated a positive correlation between serum TGF-β1 levels and left atrial voltage measured in the coronary sinus [38]. Moreover, a meta-analysis confirmed that elevated serum TGF-β1 levels are associated with an increased risk of new-onset AF [39]. Among individuals with AF undergoing CA, those exhibiting higher TGF-β1 levels tended to have more severe atrial fibrosis [40]. Another study identified serum TGF-β1 level as an independent predictor of recurrence in patients with paroxysmal AF [41]. Despite these findings, several reports have produced inconsistent results [14, 42-44], which may be attributable to the complex and heterogeneous nature of AF pathophysiology. Notably, TGF-β1 levels tend to rise during the early, paroxysmal phase of AF, whereas a diminished response is often seen in persistent AF. This shift may result from compensatory alterations in the TGF-β1 signaling pathway, possibly due to increased TGF-β1 consumption by remodeling and fibrotic atrial tissue or the antifibrotic effects of N-terminal pro-B-type natriuretic peptide [45].

Research indicates that cardiac chamber enlargement is closely associated with the extent of myocardial interstitial fibrosis—a relationship that is particularly evident in patients with left ventricular hypertrophy or left atrial dilation. For many years, researchers have attempted to quantify fibrosis as an indicator of the severity of structural remodeling. Since TGF-β plays a dominant role in fibrosis, recent studies have suggested a positive correlation between plasma TGF-β1 levels and the severity of AF. Circulating baseline TGF-β levels may serve as a potential biomarker for predicting the risk of AF recurrence after CA in the future.

## 4. TGF-β and Atrial Fibrosis

TGF-β is a member of the superfamily of secreted polypeptide growth factors, which also comprises growth and differentiation factors, activins, nodals, bone morphogenetic proteins, and anti-Müllerian hormone [46]. In mammals, TGF-β functions as a pleiotropic cytokine that regulates diverse cellular processes, including proliferation, differentiation, and apoptosis, across various cell types such as immune cells and platelets [47].

### 4.1. Activation of TGF-β

TGF-β exists in three isoforms—TGF-β1, TGF-β2, and TGF-β3 [48, 49]. Each isoform is initially synthesized in the rough endoplasmic reticulum as an inactive propeptide composed of an N-terminal signal sequence, a latency-associated peptide (LAP), and a C-terminal mature domain. After synthesis, the precursor undergoes cleavage by the protease, furin, which allows mature TGF-β fragment to non-covalently associate with LAP, resulting in the formation of small latent complex (SLC) [50]. TGF-β activation generally occurs via three mechanisms. First, SLC can interact with latent TGF-β binding protein (LTBP) within ECM, facilitating the release of mature TGF-β via interaction with α/β-integrins [51]. Certain integrins, such as integrin β3, are known to play a crucial role in this process. For instance, integrin β3 serves as a receptor involved in TGF-β1 activation. In addition, the glycoprotein milk fat globule-epidermal growth factor 8 has been demonstrated to attenuate atrial fibrosis in an angiotensin II-induced model by inhibiting the TGF-β1 signaling pathway through binding to integrin β3 [52]. Moreover, SLC can also associate with glycoprotein A repetitions predominant, a membrane protein present on regulatory T cells, platelets, and endothelial cells, promoting the activation of latent TGF-β [53]. These findings suggest that selectively inhibiting the activation of latent TGF-β may represent an effective therapeutic strategy to prevent the initiation of fibrotic remodeling.

### 4.2. Signaling Pathways of TGF-β

Once activated, TGF-β binds to a pair of transmembrane serine/threonine kinase receptors: type I (TβRI) and type II (TβRII) [54]. TGF-β signaling proceeds through the canonical (Smad-dependent) and non-canonical (Smad-independent) pathways, each contributing to cellular regulation under distinct physiological and pathological conditions.

#### 4.2.1. Canonical TGF-β Signaling Pathway

Within the canonical TGF-β signaling pathway, the active form of the ligand initially binds to the low-affinity co-receptor, TGF-β type III receptor (TβRIII). This interaction facilitates the subsequent transfer of TGF-β to the high-affinity receptor complex, which consists of TβRI and TβRII [50, 55]. Upon ligand binding, TGF-β activates TβRII, which subsequently phosphorylates the GS domain of TβRI. This phosphorylation induces a structural alteration in TβRI, allowing it to activate Smad2 and Smad3 through phosphorylation [56]. Upon phosphorylation, Smad proteins bind to Smad4, resulting in the formation of heterotrimeric complexes that translocate to the nucleus and modulate the transcription of specific target genes [57]. Of the receptor-regulated Smads, Smad3 is especially important for driving fibrotic responses via the canonical TGF-β pathway. Notably, inhibiting the expression of phosphorylated Smad3 has been linked to a decrease in fibrosis progression [58, 59]. In a rat model of AF, the transcription factor, PU.1, has been shown to enhance Smad3 activation, promoting fibrotic remodeling in cardiac tissue [60]. Furthermore, studies have demonstrated that intermedin 1-53 can inhibit Smad3 phosphorylation and reduce the expression of collagen types I and III at the mRNA and protein levels following MI in rats [61]. Prior studies have also linked atrial natriuretic peptide dysregulation to AF [62, 63]. Silencing natriuretic peptide receptor-C expression in cultured fibroblasts led to decreased Smad2 phosphorylation and diminished collagen synthesis upon stimulation with TGF-β1 [64]. Moreover, apelin, an endogenous bioactive peptide, has been reported to attenuate atrial fibrosis and decrease susceptibility to AF in Ang II-treated rats by inhibiting Smad2-driven differentiation of myofibroblasts [65]. Furthermore, activation of the retinoid X receptor α by 9-cis-RA has been reported to interact with phosphorylated Smad2 in the cytoplasm, preventing its nuclear translocation and thereby suppressing TGF-β1/Smad signaling, leading to attenuation of fibrosis in myofibroblasts [66]. Conversely, Smad7 functions as an inhibitory regulator in the TGF-β signaling pathway by competing with TβRI, serving as a negative feedback inhibitor of the Smad-dependent pathway [67]. Expression of Smad7 was observed to be significantly reduced in a rapid atrial pacing-induced animal model [68]. Additionally, the G-protein-coupled ER (GPR30) has been implicated in non-genomic estrogen signaling and cell growth regulation. A GPR30-specific agonist has been shown to increase Smad7 expression, thereby suppressing the TGF-β1/Smads pathway and alleviating atrial fibrosis in ovariectomized mice [69]. Within the nucleus, transcriptional complexes formed by Smad2, Smad3, and Smad4 participate in the regulation of downstream gene expression [70]. Together, these findings underscore the critical involvement of the canonical signaling cascade in fibrosis development.

#### 4.2.2. Non-Canonical TGF-β Signaling Pathway

Apart from Smad-dependent signaling, TGF-β also activates non-canonical pathways such as MAPK, PI3K/Akt, NF-κB, and JAK/STAT, which collectively contribute to the progression of cardiac fibrosis through downstream signal modulation [71-73]. For instance, inhibition of p38-MAPK suppresses TGF-β-induced myofibroblast activation and limits excessive ECM deposition, whereas its overexpression promotes myofibroblast differentiation [74]. Moreover, studies in mouse myofibroblasts have shown that TGF-β1 upregulates the expression of connexin 43 (Cx43) protein, which may partially explain the heightened Cx43 levels detected in fibroblasts derived from infarcted cardiac tissue [75]. Decreased expression of Cx43 has been associated with impaired intercellular electrical coupling, contributing to the onset and progression of AF. Additionally, C1q/tumor necrosis factor-related protein 9 (CTRP9), a cardiac-enriched member of the CTRP superfamily, is abundantly expressed in myocardial tissue. Recent studies suggest that CTRP9 reduces early stage AF following MI by inhibiting arrhythmogenic processes, such as inflammation and fibrosis, which is related to the suppression of the TLR4/NF-κB signaling pathways [76]. During cardiac stress, vascular peroxidase1 is activated by elevated TGF-β1 levels, subsequently stimulating the HOCl/ERK1/2 signaling pathways, thereby promoting myofibroblast differentiation and proliferation [77]. Furthermore, ubiquitin-specific protease 7 (USP7), a deubiquitinating enzyme known to regulate cell proliferation, has been implicated in the pathophysiological processes underlying AF [78]. Inhibition of USP7 has been shown to attenuate Ang II-induced activation of NF-κB/NLRP3 and NADPH oxidases signaling. Additionally, USP7 suppression increases Cx43 expression while decreasing levels of SERCA2a, oxidized CaMKII, total CaMKII, and Kir2.1—all of which are associated with the initiation and progression of AF [78]. These non-Smad pathways add complexity and regulatory control to the TGF-β signaling network, enabling diverse cellular responses to TGF-β stimulation.

### 4.3. Mechanisms of TGF-β-Mediated Atrial Fibrosis

Evidence from preclinical studies and clinical observations indicates that TGF-β isoforms, especially TGF-β1, function as key mediators in the development of organ fibrosis [79, 80]. Among the various mechanisms, post-translational modulation of TGF-β signaling is closely linked to the regulation of fibrogenic gene expression and plays a critical role in the onset and progression of fibrosis [54]. TGF-β activation induces fibroblast differentiation into contractile myofibroblasts. Activation of fibroblasts results in increased cellular and nuclear size, enhanced protein synthesis, and concomitant changes in metabolism and gene expression [81, 82]. Galectin-3 (Gal-3), a β-galactoside-binding lectin primarily secreted by macrophages, has been recognized as an important mediator of inflammation and angiogenesis [83]. Research involving cultured rat atrial fibroblasts has revealed that Gal-3 activation in atrial tissue promotes TGF-β1 signaling, leading to elevated levels of α-SMA and collagen I, which may accelerate the development of atrial fibrosis [84]. In addition, clinical studies suggest that pharmacological inhibition of Gal-3 could be used as a supplementary approach to improve CA effectiveness in individuals with persistent AF [85].

Recent studies have highlighted the role of fibroblast-like cells originating from endothelial cells via endothelial-to-mesenchymal transition (EndMT) in the pathogenesis of cardiac fibrosis. In human endocardial cells, TGF-β1 has been shown to induce EndMT, at least partly, through the upregulation of miR-181b expression [86]. Recent research has highlighted the involvement of non-coding RNAs as important regulators in the pathogenesis of cardiac fibrosis [71, 87-91]. This process occurs through the Smad2/3 signaling pathway [86]. Additionally, omentin-1, a protein abundantly expressed in human visceral adipose tissue, regulates endothelial function [92]. It has been reported to suppress Smad3 phosphorylation, normalize the expression of vimentin and VE-cadherin, inhibit the TGF-β1/Smad3 signaling cascade, and ultimately block the process of EndMT [93].

In addition, TGF-β contributes to atrial fibrosis by interacting with CD44, a transmembrane glycoprotein that serves as the principal receptor for hyaluronan. In a tachypacing-induced atrial myocyte model, Yeh et al. demonstrated that CD44 plays a critical role in atrial remodeling [94, 95]. In TGF-β transgenic mice, TGF-β-mediated CD44/STAT3 signaling was observed to promote atrial fibrosis and AF, primarily through increased extracellular collagen deposition in the atria [96]. In contrast, TGF-β1 has also been shown to induce CD39 expression, which forms a negative feedback mechanism that suppresses myofibroblast activation and mitigates fibrosis in vivo after MI. This regulatory effect contributes to reduced collagen deposition and preservation of elastin content [97].

TGF-β has recently been found to mediate its profibrotic actions in fibroblasts through a mechanism that depends on interleukin-11 (IL11). Notably, TGF-β significantly increases IL11 expression in human and mouse myofibroblasts, underscoring the importance of this downstream signaling axis in fibrotic remodeling [98]. In addition, YAP and TAZ, which function as key downstream mediators of the Hippo signaling pathway, are critical for promoting cardiomyocyte proliferation during cardiac development and tissue repair. Recent studies indicate that YAP and TAZ also contribute to organ fibrosis by promoting the transformation of fibroblasts into myofibroblasts, thereby accelerating collagen overproduction and fibrotic progression [99]. In myocardial fibrosis, YAP acts as a downstream integrator of the Wnt and TGF-β signaling pathways. Its activation in myofibroblasts has been associated with an increased secretion of the profibrotic cytokine, interleukin-33, further contributing to fibrotic remodeling within cardiac tissues [100]. Notably, in a rat model of myocardial injury, a nanofibrous cardiac patch incorporating reduced graphene oxide and silk fibroin significantly alleviated cardiac fibrosis and improved cardiac performance. These therapeutic benefits were mechanistically linked to pronounced suppression of the YAP/TAZ-TGF-β1/Smads signaling cascade in the treated group [101].

**Figure 2.**
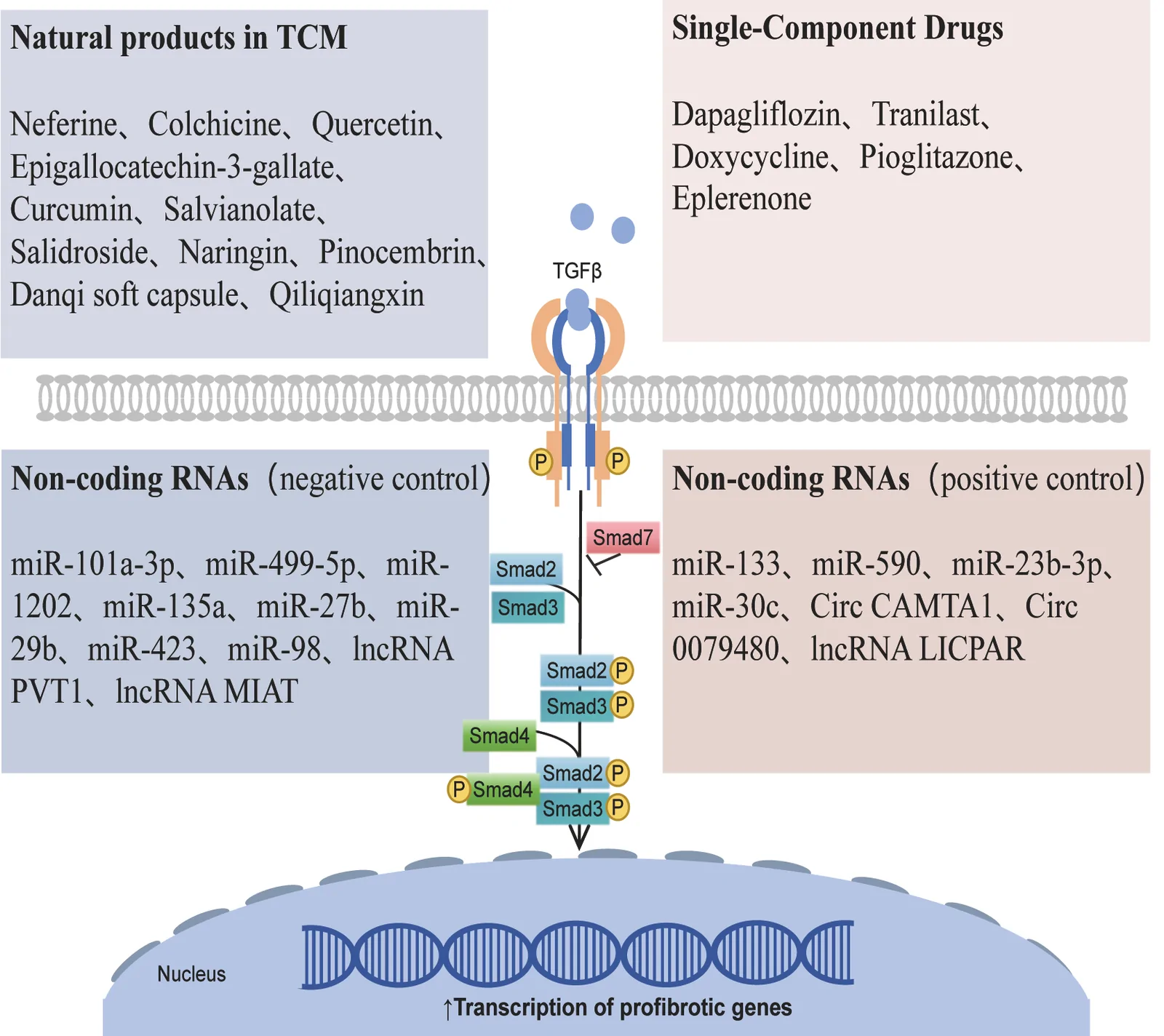
Regulation of canonical TGF-β signaling modulators as an alternative therapeutic approach in AF.

In conclusion, TGF-β-driven fibrosis is mediated through the Smad-dependent and non-Smad pathways, with its effects further regulated by coreceptors and interacting signaling networks, as shown in Figure 2. Upon activation, TGF-β binds to its receptors, initiating a cascade of phosphorylation events that activate Smad2, Smad3, and Smad4. Following phosphorylation, Smad proteins enter the nucleus, where they interact with specific transcription factors and cofactors to initiate transcription of genes involved in fibrosis. Concurrently, TGF-β-induced EndMT and signaling mediated by CD44 have also been implicated in the pathogenesis of atrial fibrosis associated with AF. Accordingly, targeting the TGF-β signaling cascade with pharmacological inhibitors at the early phase of structural remodeling may suppress fibrotic progression and mitigate arrhythmogenic remodeling, presenting a viable therapeutic strategy for AF management.

## 5. Preclinical Strategies of TGF-β Inhibition in AF

As discussed earlier, TGF-β signaling is fundamentally involved in the progression of fibrosis, operating through multiple distinct molecular mechanisms, including activation of resident fibroblasts, promotion of apoptotic pathways, and induction of EndMT. In response, extensive research has focused on mitigating these pathological changes by targeting the canonical TGF-β signaling pathway, as depicted in Figure 2. The subsequent subsections provide an overview of current therapeutic approaches designed to inhibit TGF-β signaling for the management of AF, with relevant strategies detailed in Table 1.

### 5.1 Non-Coding RNAs (ncRNA)-Based Therapies

ncRNAs are typically categorized as miRNAs, long non-coding RNAs (lncRNAs), and circular RNAs (circRNAs) according to their functional properties [102]. Studies have uncovered complex regulatory networks underlying cardiac fibrosis that involve interactions among lncRNAs, miRNAs, and histone post-translational modifications. Notably, these molecules exert their profibrotic influence primarily by modulating the TGF-β/Smad signaling pathway [103].

#### 5.1.1. miRNAs

Current research demonstrates that miRNAs are crucial regulators of cardiac fibrosis, ion channel activity, calcium homeostasis, and inflammatory responses—all of which are involved in the pathogenesis of AF [104, 105] Over the past 5 years, miRNAs have emerged as important epigenetic regulators that contribute to the development and progression of cardiac fibrosis. They exert their effects by directly influencing the transcription and translation of genes implicated in fibrosis, often in coordination with ncRNAs that modulate gene expression at various regulatory levels. Specific miRNAs can enhance or suppress the expression of fibrosis-related genes through precise transcriptional control mechanisms [87, 106].

Upregulating antifibrotic miRNAs is considered a promising strategy for alleviating cardiac fibrosis and lowering the incidence of AF. In a nicotine-induced canine model of AF, decreased levels of miR-133 and miR-590 were associated with elevated expression of TGF-β1 and its receptor, TβRII. Conversely, transfection of miR-133 or miR-590 into cultured atrial fibroblasts suppressed TGF-β1 and TβRII levels and led to a reduction in collagen deposition [107]. Furthermore, upregulation of miR-101a-3p has been demonstrated to inhibit the expression of several profibrotic markers, such as TGF-β1, connective tissue growth factor (CTGF), fibronectin, and α-SMA. Zhu et al. found that miR-101a-3p reduces atrial fibrosis and prevents the onset of AF in acetylcholine-CaCl_2_-induced rat models, partly through the inhibition of enhancer of zeste homolog 2 [108]. In patients with mitral stenosis, miR-1202 exhibited the highest level of upregulation among 28 analyzed miRNA in AF than in sinus rhythm [109]. Prior research indicates that miR-1202 can activate the TGF-β1/Smads signaling pathway and simultaneously suppress cardiac fibrosis in fibroblasts by targeting neuronal nitric oxide synthase [110]. Although miR-135a has mainly been studied in the context of oncology, emerging research has identified its involvement in AF, where persistent downregulation of miR-135a is observed alongside atrial fibrosis in both in vivo and in vitro models. MiR-135a exhibits antifibrotic effects through the Smad3/TRPM7 signaling axis in AF [111]. Similarly, increased expression of miR-29b has been shown to mitigate atrial fibrosis; decrease COL1A1, COL3A1, and TGF-β1 levels; and shorten the duration of AF episodes [112]. Additional evidence suggests that miR-30c is a key modulator of cardiac fibrosis [113]. Elevated miR-30c suppresses TβRII expression in myofibroblasts, thereby reducing collagen production and atrial fibrogenesis. Administration of exogenous miR-30c has been proposed as a promising therapeutic approach for AF [36]. Additionally, Xiao et al. demonstrated that hsa-miR-4443 suppresses the proliferation of myocardial fibroblasts in AF by targeting thrombospondin-1, thereby modulating the TGF-β1/α-SMA/collagen signaling pathway [114]. MiR-499-5p, a member of the miRNA-499 family that is highly expressed in cardiomyocytes, has been shown to play a regulatory role in multiple cardiac conditions. Recent findings indicate that miR-499-5p inhibits the proliferation, migration, and collagen synthesis of atrial fibroblasts by targeting TβRI and downregulating TGF-β1/Smad2 signaling at the mRNA and protein levels [115].

**Table 1:** Preclinical strategies of TGF-β in AF.

Targeting therapy		Experimental Model	Type	Targets
**ncRNA-based Therapies**	MiR-13 [107]	Nicotine-induced dogs; atrial fibroblasts from dogs	Both	miR-133, miR-590inhibits TGF-β1, TβRII
	MiR-590 [107]			
	MiR-101a-3p [182]	Acetylcholine-CaCl_2_-induced rats	In vivo	miR-101a-3p inhibits TGF-β1
	MiR-1202 [110]	Human myofibroblasts	In vitro	miR-1202 promotesTGF-β1, p-Smad2/3
	MiR-135a [111]	Rats induced by acetylcholine-CaCl_2_; Rat atrial fibroblasts	Both	miR-135a promotes Smad3
	MiR-29b [183]	Rats induced by Ach-CaCl_2_;	In vivo	miR-29b inhibitsTGFβ1, TβRI, p-Smad2/3
	MiR-30c [184]	Rats induced by abdominal aortic constriction; myofibroblasts	Both	miR-30c inhibits TβRII
	MiR-4443 [185]	Human myofibroblasts	In vitro	hsa-miR-4443inhibits TGF-β1
	MiR-499-5p [186]	Atrial fibroblasts	In vitro	miR-499-5p inhibits TGFβ1, TβRI, Smad2
	MiR-27b [116]	Mice induced by Ang II	In vivo	miR-27b inhibits p-Smad2/3
	MiR-23b-3 [187]	Human Atrial Fibroblasts	In vitro	miR-23b-3p, miR-27b-3ppromotes p-Smad3, and inhibitsTGFBR3, p-Smad1
	MiR-27b-3p [187]			
	MiR-27b-3p [105]	Rats induced by Ach-CaCl2;	In vivo	miR-27b-3p inhibits TGF-β1
	MiR-21 [188]	Atrial tachypacing-induced rabbit model; TGF-β1-induced adult rat myofibroblasts	Both	MiR-21 inhibits Smad7
		Human myofibroblasts	In vitro	miR-21 promotesTGF-β1,p-Smad2
	Circ 0079480 [189]	C57/B6J mice induced by Ang II; Human atrial fibroblasts	Both	circ_0079480 promotes TGF-β1, p-Smad3
	Circ CAMTA1 [190]	Human atrial fibroblasts; Mouses induced by AngII	Both	circCAMTA1 promotes TβRI
	LncRNA PVT1 [134]	C57BL/6J mice induced by Ang II; Human atrial fibroblasts	Both	LncRNA PVT1 inhibits TGF-β1, p-Smad2, p-Smad3
	LncRNA LICPAR [191]	Human Atrial Fibroblasts	In vitro	LIPCAR promotes TGF-β1, p-Smad2, p-Smad3
	LncRNA MIAT [192]	SD rats induced by electrical stimulation; Atrial tissue from AF rats	Both	MIAT promotes TGF-β
**Single-Component Drugs**	Dapagliflozin [193]	Mouses induced by Ang II; HL-1 and primary mouse fibroblast;	Both	Dapaglifloz ininhibits TGF-β1, p-Smad2/3
	Doxycycline [194]	Chronic intermittent hypoxia rat model	In vivo	Doxycycline inhibits TGF-β1, TβRI/II, p-Smad2/3
		Chronic intermittent hypoxia rat model	In vivo	Doxycycline inhibits TGF-β1
	Pioglitazone [195]	Alloxan-induced diabetic rabbits	In vivo	Pioglitazone inhibits TGF-β1
**Natural Compounds**	Neferine [147]	C57BL/6 mice induced by Ang II; HL-1 cells	Both	Neferine inhibits TGF-β1, p-Smad2, p-Smad3
	Colchicine [196]	Rats induced by Ach-CaCl2	In vivo	Colchicine inhibits TGF-β1
		SD rats induced by transesophageal burst pacing; Rat myofibroblasts	Both	Colchicine inhibits TGF-β
	Quercetin [197]	Rats treated with ISO; Rat myofibroblasts induced by ISO	Both	Quercetin inhibits TGFBR1/2, Smad2/3/4;
	Epigallocatechin-3-gallate [198]	SD rats induced by Ang-II and transesophageal burst pacing; Rat myofibroblasts	Both	Epigallocatechin-3-gallate inhibits TGF-β1, Smad3/p-Smad3
	Curcumin [199]	Rats induced by Ach - CaCl2	In vivo	Curcumininhibits TGF-β1,
	Salvianolate [200]	SD rats induced by LAD;	In vivo	Salvianolate inhibits TGF-β1, Smad2/3
	Salidroside [165]	Male C57BL/6 mice induced by Ang II and transesophageal burst pacing	In vivo	Salidrosideinhibits TGF-β1, p-Smad2, p-Smad3
	Naringin [201]	MHC-TGF-Cys33Ser transgenic mice; Human atrial tissue samples; Human atrial endocardial endothelial cells; Human myofibroblasts;	Both	Naringininhibits TGF-β
	Pinocembrin [202]	SD rats induced by ISO	In vivo	Pinocembrin inhibits TGF-β
		SD rats treated with chronic unpredictable mild stress	In vivo	Pinocembrin inhibits TGF-β1
	Danqi soft capsule [203]	Rats induced by LAD ligaton; myofibroblasts	Both	Danqi soft capsule inhibits TGF-β1, p-Smad3
	Qiliqiangxin [204]	Rabbits	In vivo	Qiliqiangxin inhibits TGF-β1, p-Smad2/3

Suppression of pro-fibrotic gene expression can hinder the progression of fibrosis in AF. For instance, miR-27b, which is primarily expressed in the left atrium and belongs to the miR-27 family, has been reported to promote the expression of genes associated with fibrosis in various experimental settings [105, 116, 117]. Yang et al. reported that the levels of miR-23b-3p and miR-27b-3p were significantly upregulated in patients with AF and in vitro models of Ang-II-stimulated human atrial fibroblasts. Notably, this upregulation contributed to increased collagen production and promoted atrial fibrosis by targeting the TGF-β1 receptor, subsequently activating the Smad3 signaling pathway [117]. Several studies have demonstrated that miR-21 plays a pivotal role in regulating atrial structural remodeling. Its overexpression promotes fibrosis by activating the STAT3 signaling pathway, partly through the downregulation of CADM1 expression [118-120]. Additionally, reduced expression of Smad7 has been implicated in the progression of atrial fibrosis in AF. He et al. found that elevated miR-21 enhances the TGF-β1/Smad signaling pathway by inhibiting Smad7, whereas transfection with a miR-21 inhibitor led to increased Smad7 levels and decreased expression of collagen types I and III [121]. Furthermore, miR-21 suppresses myofibroblast proliferation by upregulating WW domain-containing protein 1, which subsequently inhibits the TGF-β1/Smad2 signaling pathway [122].

#### 5.1.2. circRNAs

circRNAs are essential regulatory molecules that modulate transcription by directly binding to the target miRNAs, thereby influencing gene expression [123, 124]. Gao et al. identified circ_0004104 in blood samples from patients with persistent AF and demonstrated that it promotes cardiac fibrosis by activating the TGF-β signaling pathway [125]. Zhang et al. identified 14,215 circRNAs in samples from patients with AF and healthy controls, proposing that specific circRNAs, including hsa_circ_0000075 and hsa_circ_0082096, contribute to the pathogenesis of AF through modulation of the TGF-β signaling pathway [126]. For instance, circ_0079480 promotes the expression of THBS1 by sponging miR-338-3p, leading to the activation of the TGF-β1/Smad3 signaling pathway. This process stimulates the proliferation, migration, and fibrosis of atrial fibroblasts in atrial fibroblasts derived from humans and Ang II-treated mice [127]. Additionally, circRNA calmodulin binding transcription activator 1 regulates the miR-214-3p/TβRI axis, thereby facilitating Ang II-induced atrial fibrosis [128]. These circRNAs act as key regulators in the development of cardiac fibrosis, often serving as negative modulators in the progression of AF. Furthermore, they hold potential as diagnostic and prognostic biomarkers. Recently, Yang et al. reported that elevated plasma levels of hsa_circ_0099734 were associated with an increased risk of AF, as well as stroke and all-cause mortality in patients with AF [129].

#### 5.1.3. lncRNAs

lncRNAs act as competitive endogenous RNAs, binding to miRNAs and preventing their interaction with target mRNAs [130]. Several lncRNAs, including lncRNA H19, lncRNA myocardial infarction-associated transcript (MIAT), and lncRNA predicting cardiac remodeling (LIPCAR), have been involved in the regulation of cardiac fibrosis [131-133]. For instance, upregulation of LIPCAR enhances the profibrotic effects of Ang II, such as increasing the expression of p-Smad2/3, collagen I, collagen III, and α-SMA, while also promoting atrial fibroblast viability and proliferation. Conversely, LIPCAR silencing produces the opposite effects [133]. Furthermore, LncRNA PVT1 can negatively modulate the function of miR-128-3p, leading to the upregulation of its targeted mRNAs, which subsequently activates the TGF-β1/Smad signaling pathway to regulate fibrosis [134]. Similarly, lncRNA MIAT has been shown to influence the expression of TGF-β1, collagen I, collagen III, and CTGF in AF rat models [135].

Nevertheless, to date, no ncRNAs regulating myocardial fibrosis via the TGF-β signaling pathway have been assessed in clinical trials. Moreover, therapeutic agents targeting TGF-β have been predominantly confined to oncology, particularly immuno-oncology. Furthermore, safety concerns arising from preclinical animal studies, such as off-target effects and immune-related toxicities, have limited their translation into clinical application for fibrotic cardiovascular diseases [136]. Moreover, the widespread distribution of ncRNAs across various cell types, tissues, and biological fluids raises concerns regarding potential off-target effects. Under normal physiological conditions, TGF-β signaling is crucial for various biological processes, including embryonic development, wound healing, tissue homeostasis, and immune system regulation. Notably, preclinical studies have indicated that blocking TβRI leads to an increased occurrence of hemorrhagic, degenerative, and inflammatory lesions in cardiac valves [94]. Additionally, inhibition of TGF-β signaling has been linked to the onset of chronic inflammatory responses in the skin and gastrointestinal tract, which may further contribute to the development of precancerous lesions.

### 5.2. Single-Component Drugs

Dapagliflozin (DAPA), a selective SGLT2 inhibitor, is commonly used to improve clinical outcomes in individuals with type 2 diabetes and heart failure. Studies have shown that DAPA improves structural remodeling by modulating the TGF-β1/Smad signaling pathway, independently of its glucose-lowering effects [137]. Zhan et al. demonstrated that DAPA inhibits the TGF-β1/Smad pathway and reduces the expression of key proteins involved in electrical activity, such as Nav1.5, Kv4.3, Kv4.2, Kchip2, Kir2.1, and connexin 40 (Cx40), in an angiotensin II-induced animal model, ultimately decreasing the incidence of AF [138].

Tranilast, a common antiallergic drug, has also demonstrated potential as an anti-fibrotic agent. S100A11, a member of the S100 protein family, has been identified through differential proteomic analysis as a novel regulator of collagen synthesis in human myofibroblasts [139]. In Ang II-induced myocardial fibrosis models, Tranilast has been shown to reduce fibrotic progression by inhibiting the S100A11/TGF-β1/ Smads signaling pathway [140].

Doxycycline has been shown to markedly reduce atrial fibrosis, conduction abnormalities, and AF susceptibility in a chronic intermittent hypoxia rat model, primarily by modulating the expression of TGF-β1, TβRI, TβRII, phosphorylated Smad2/3, α-SMA, CTGF, and collagen [141]. Furthermore, in vitro experiments using chronic intermittent hypoxia have demonstrated that doxycycline treatment can significantly decrease the severity of atrial fibrosis and the incidence of AF [142].

Pioglitazone, an oral antidiabetic agent of the thiazolidinedione class, has been reported to mitigate atrial remodeling and reduce AF occurrence. In alloxan-induced diabetic rabbit models, pioglitazone has been shown to decrease TGF-β1 expression, along with reductions in ERK2, phosphorylated ERK, Toll-like receptor 4, NF-κB p50, and heat shock protein levels [143].

Eplerenone, a selective mineralocorticoid receptor antagonist, is primarily known for its mechanism of action. However, it has also been reported to exhibit strong binding affinity for Kv1.3 potassium channels located on T lymphocytes. By directly inhibiting Kv1.3 channels, eplerenone reduces the proliferation of regulatory T cells, which are responsible for secreting TGF-β, thus playing a role in the suppression of atrial fibrosis [144-146].

Accumulating evidence suggests that enhanced TGF-β signaling consistently serves as a dominant driver of organ fibrosis under various pathological conditions including inflammation, hypoxia/reoxygenation, and metabolic disorders. Consequently, angiotensin II receptor blockers, anti-inflammatory agents, angiotensin-converting enzyme inhibitors, and other pharmacological compounds not primarily classified as AADs may hold therapeutic promise for the management of AF and associated fibrotic remodeling.

### 5.3. Natural Compounds

#### 5.3.1. Alkaloids

Neferine, a bioactive compound derived from the seeds of *Nelumbo nucifera Gaertn*, has been shown to activate the Nrf2/HO-1 signaling pathway while simultaneously inhibiting the TGF-β/Smads pathway. These actions contribute to a reduction in the onset and severity of AF, as well as the extent of atrial fibrosis [147]. Additionally, Neferine partially reverses elevated oxidative stress levels [147]. Colchicine, the mostly studied compound derived from the colchicine plant, plays a protective role in cardiovascular diseases [148]. Recent findings suggest that colchicine may exert antifibrotic effects on the myocardium, thereby contributing to a reduction in the incidence of AF. One study reported that colchicine treatment shortened the duration of AF and alleviated left atrial fibrosis, and these effects correlated with a significant decrease in TGF-β1 expression. Furthermore, colchicine inhibited the secretion of activin A, collagen type I, and collagen type III, suggesting its potential as a therapeutic agent for AF [149].

#### 5.3.2. Terpenoids

Zerumbone, a compound isolated from *Syringa pinnatifolia Hemsl*, exhibits various biological effects including antimicrobial, antioxidant, anti-inflammatory, antitumor and antihyperalgesic effects [150]. In a MI model, treatment with zerumbone significantly enhanced cardiac functional parameters, such as ejection fraction and fractional shortening. In vitro studies conducted using cardiac myofibroblasts further showed that zerumbone reduced the expression of α-SMA, TGF-β1, p-Smad2/3, and matrix metalloproteinases, thereby mitigating cardiac fibrosis [151].

#### 5.3.3. Flavonoids

Quercetin, a flavonoid commonly found in a variety of fruits and vegetables, has been shown to exhibit cardioprotective effects in a rat model of isoproterenol (ISO)-induced cardiac injury [152]. In atrial tissues from AF models, the expression of miR-135b was observed to be decreased. Quercetin treatment alleviated AF by upregulating miR-135b expression, primarily through the inhibition of the TGF-β/Smads signaling pathway. This regulatory effect resulted in the downregulation of genes and proteins associated with collagen synthesis, which are involved in the progression of atrial fibrosis [153].

Pinocembrin, a flavanone with potent anti-inflammatory and antioxidant effects, has been demonstrated to protect against myocardial ischemia-induced cardiac fibrosis and arrhythmias in preclinical models [154]. Yang et al. reported that pinocembrin administration attenuated myocardial fibrosis through the downregulation of TGF-β and fibrosis-related proteins, including collagen I, collagen III, and α-SMA, as well as upregulation of Cx40 [155]. Moreover, in a rodent model of depression, pinocembrin was found to inhibit the pro-apoptotic p-p38 MAPK signaling and TGF-β1-mediated pro-fibrotic pathways [156].

#### 5.3.4. Polyphenols

Epigallocatechin-3-gallate (EGCG), a key bioactive compound found in tea polyphenols, has been shown to reduce Ang II-induced cardiac fibrosis, primarily by inhibiting the TGF-β/Smad3 signaling pathway. This inhibition results in the downregulation of lysyl oxidase, an enzyme responsible for the oxidative deamination of lysine and hydroxylysine residues in collagen fibers, thus contributing to the formation of fibrotic scars. Consequently, EGCG decreases collagen deposition, thereby inhibiting the development of AF [157]. In addition, previous animal studies have suggested that EGCG directly modulates electrophysiological properties and Ca^2+^ homeostasis in ISO-induced models by inhibiting Ca^2+^/calmodulin and cyclic guanosine monophosphate-dependent protein kinase. This regulation, in turn, suppresses atrial arrhythmogenesis [158]. Curcumin, a bioactive compound found in turmeric and *Curcuma xanthorrhiza* oil, has been shown to decrease the secretion of proinflammatory cytokines, including IL-17A, IL-1β, IL-6, and TGF-β1, indicating its potential in reversing myocardial fibrosis [159]. Similarly, resveratrol, a polyphenolic compound derived from various plants, has been widely recognized for its antifibrotic effects across multiple organs. In both in vivo and in vitro diabetes models, Liu et al. found that resveratrol administration reduced collagen synthesis, mainly through the TGF-β1/Smad3 signaling pathway, indicating resveratrol’s therapeutic potential for cardiac fibrosis associated with hypertension and diabetes [160]. Salvianolate, the most extensively studied bioactive constituent of *Salvia miltiorrhiza Bunge*, has been widely recognized for its cardioprotective properties. Evidence suggests that salvianolate administration may reduce the incidence of AF by inhibiting the TGF-β1/Smads signaling pathway and TXNIP/NLRP3 inflammasome cascade, thereby mitigating atrial interstitial fibrosis [161].

#### 5.3.5. Glycoside

Lysyl oxidase-like 2 (LOXL2), a multifunctional enzyme secreted into the extracellular matrix, facilitates collagen cross-linking and matrix stabilization [162]. Clinical studies have demonstrated that serum LOXL2 levels are significantly elevated in patients with AF and positively correlate with the extent of left atrial fibrosis [163]. Therefore, increased LOXL2 levels may serve as a biomarker for left atrial structural remodeling and hold potential for predicting AF severity. LOXL2, a copper-dependent amine oxidase, is implicated in various fibrosis-associated diseases. Inhibition of LOXL2 has been shown to attenuate cardiac fibrosis, cardiac hypertrophy, and inflammation. These effects are associated with a decreased expression of TGF-β1, p-Smad2/3, and collagen I in atrial tissue [164]. Salidroside, an active component derived from *Rose rhodiola*, has been demonstrated to suppress atrial fibroblast apoptosis and oxidative stress while downregulating LOXL2 levels and inhibiting the TGF-β1/Smad signaling pathway in AF model [165]. Semaphorin-3A (Sema3A) is a membrane-associated protein that regulates many cellular processes, and its downregulation is associated with EndMT in atrial fibrosis. Naringin, a flavanone glycoside, functions as a Sema3A activator by upregulating its expression. A recent study demonstrated that naringin enhances Sema3A expression, thereby suppressing TGF-β-induced EndMT in vitro. Furthermore, naringin administration has been shown to ameliorate AF and endocardial fibrosis in TGF-β transgenic mice [166].

#### 5.3.6. Herbal Remedies

Danqi soft capsule, a complementary and alternative Chinese herbal preparation composed of *Salvia miltiorrhiza* and *Panax notoginseng*, has demonstrated therapeutic potential in AF. In a rat model of heart failure following MI, this formulation improved left atrial arrhythmogenic substrate by suppressing myofibroblast proliferation, migration, collagen production, and differentiation [167].

Qiliqiangxin, a traditional Chinese herbal formulation commonly used in the treatment of chronic heart failure, has been shown to alleviate atrial structural remodeling in a rabbit model of prolonged pacing-induced AF. This effect is achieved through the inhibition of the TGF-β1/Smad2/3 signaling pathway [168].

Huoxin Pill, a traditional Chinese herbal formulation commonly used for the treatment of myocardial ischemic diseases, has been shown to reduce myofibroblast differentiation and collagen synthesis in rats with ISO-induced heart injury. This effect is associated with the downregulation of elevated TGF-β1 and phosphorylated Smad2/3 levels, thereby exerting a protective effect against myocardial fibrosis [169].

Emerging evidence has established a causal link between increased TGF-β activity and the initiation as well as progression of fibrosis. Several animal models have demonstrated that active compounds derived from single herbs and traditional Chinese medicine formulas, particularly flavonoids, glycosides, and alkaloids, exhibit potential in mitigating atrial fibrosis associated with AF. However, further research is needed to clarify the unidentified chemical compositions and underlying regulatory mechanisms involved, conduct standardized clinical trials, and assess potential side effects.

### 5.4. Non-Drug Therapies

Previous studies have established a correlation between prolonged or excessive exercise training and an elevated risk of AF [170, 171]. In a rat model subjected to long-term intensive exercise training, elevated expression levels of TGF-β1 were observed [172]. In a transgenic goat model overexpressing TGF-β1, endurance exercise performed in the presence of preexisting atrial myopathy was found to increase the susceptibility to AF [173]. Conversely, exercise training has been shown to modulate the TGF-β1/Smad2/3/MMP2/9 signaling pathway, leading to a reduction in cardiac fibrosis in murine models of MI [174]. Additionally, irisin, a hormone induced by exercise, has been reported to alleviate neuro-inflammation and mitigate neurodegeneration [175]. A study demonstrated that irisin inhibits atrial fibrosis and AF development by inactivating LOXL2 and suppressing the TGFβ1/Smad2/3 signaling pathway [176]. Moreover, resistance exercise has been shown to upregulate the expression of irisin, which plays a role in reducing post-MI cardiac fibrosis. This effect is mediated through the activation of the AMPK/SIRT1 signaling pathway and concurrent inhibition of the TGF-β1/Smad2/3 axis [177].

Clinical observations frequently highlight the coexistence of AF and left ventricular dysfunction [178]. In a canine model with healed MI, chronic vagus nerve stimulation was found to alleviate left ventricular interstitial fibrosis, accompanied by significant reductions in TGF-β1, collagen I, collagen III, and MMP9 expression in the left ventricular tissues [179].

Collectively, these findings suggest that endurance exercise and vagus nerve stimulation may represent promising non-pharmacological interventions targeting the TGF-β signaling pathways for mitigating inflammation and fibrosis in patients with AF.

## 6. Conclusion and Future Outlook

With the aging rapidly global population, the prevalence of AF is expected to increase significantly by 2050. Atrial fibrosis has emerged as a key pathological substrate that not only contributes to the initiation but also accelerates the progression of AF. However, current therapeutic approaches for AF are constrained by factors such as the increased risks of cardiac adverse events, hemorrhagic stroke, and drug-induced gastrointestinal bleeding. Consequently, targeting cardiac fibrosis offers a promising therapeutic strategy for managing AF. TGF-β is widely recognized as a central regulator of fibrotic remodeling, and interventions that modulate TGF-β signaling are increasingly viewed as a potential upstream approach for the prevention and treatment of AF.

Emerging data suggest that single-component drugs including Tranilast and Pioglitazone, as well as natural bioactive compounds such as quercetin, curcumin, salvianolate, and naringin, may exert antifibrotic effects by modulating the TGF-β signaling cascade. However, their translation into effective clinical interventions remains limited. Lewis et al. found that administration of pirfenidone, an inhibitor of TGF-β signaling, resulted in decreased myocardial fibrosis in patients with heart failure with preserved ejection fraction, as evidenced by the clinical trial NCT02932566 [180]. Another clinical trial (NCT00011076) evaluated the efficacy of TGF-β inhibitors in reducing cardiac fibrosis among patients with hypertrophic cardiomyopathy and impaired left ventricular diastolic function, though the findings have not yet been made publicly available. Nonetheless, these investigations offer important insights that could inform the development of innovative therapeutic approaches aimed at mitigating cardiac fibrosis and, consequently, alleviating the clinical burden of AF.

Recent research has underscored the importance of transcriptome-level epigenetic changes in promoting fibrosis, which has deepened our understanding of the molecular basis of cardiac fibrosis in AF. Increasing evidence suggests that miRNAs are involved in regulating cardiac fibrotic remodeling and represent promising targets for therapeutic interventions. Nonetheless, the clinical application of epigenetic therapies remains challenging due to issues such as poor delivery efficiency, unintended effects on non-target genes, and possible immune responses. Consequently, only a limited number of epigenetic drugs have reached clinical use thus far [181]. As previously discussed, TGF-β is essential for coordinating normal tissue repair. A lack or imbalance of TGF-β signaling can compromise wound healing, cause vascular defects, disrupt immune function, and elevate the risk of tumorigenesis. Systemic blockade of TGF-β signaling can lead to adverse effects on healthy tissues. For instance, a widespread use of anti-TGF-β therapies may hinder the regeneration of liver and kidney cells, disrupt gastrointestinal mucosal healing, and cause gastrointestinal dysfunction. Given TGF-β’s central role in tissue repair following injury, novel therapeutic approaches are being developed to selectively inhibit the profibrotic or prometastatic pathways of TGF-β signaling. Techniques such as nanoparticle-mediated delivery and targeted drug administration may help limit off-target actions and decrease systemic side effects.

Moreover, in most cases, single-component drugs will not produce optimal therapeutic results, and even if there is an initial response, resistance is likely to develop. Fortunately, natural products show great advantages in dealing with this complexity. In addition, these natural products, owing to their multifaceted mechanisms, exhibit diverse effects in addition to antiarrhythmic properties, such as metabolic modulation, anti-inflammatory effects, and antioxidant effects. Adjuvant therapy with natural products alongside current protocols may be beneficial in several ways, including reducing adverse effects, overcoming drug resistance, and improving therapeutic responses. In addition, compounded herbal prescriptions were previously limited by the complexity of the active ingredients. With the development of artificial intelligence technologies, machine learning-based bioactivity and mechanism prediction may eventually provide a solution to the complexity of the traditional herbal combinations and plant extracts. Furthermore, integrative traditional Chinese medicine-based therapeutic strategies represent a valuable direction for natural product development in the treatment of AF.
